# Nutritional interventions to prevent retinopathy of prematurity

**DOI:** 10.1038/s41390-024-03208-1

**Published:** 2024-04-29

**Authors:** Ann Hellström, Elsa Kermorvant-Duchemin, Mark Johnson, Miguel Sáenz de Pipaón, Lois E. Smith, Anna-Lena Hård, on behalf of ESPR Nutrition council members, Christoph Fusch, Silvia Iacobelli, Mark J. Johnson, Alexandre Lapillonne, Sissel J. Moltu, Miguel Sáenz de Pipaón, Gitte Zachariassen

**Affiliations:** 1 Department of Clinical Neuroscience, Institution of Neuroscience and Physiology, Sahlgrenska Academy, University of Gothenburg, Gothenburg, Sweden; 2 Université Paris Cité, AP-HP, Hôpital Necker-Enfants Malades, Department of Neonatal Medicine, Paris, 75015 France; 3 Department of Neonatal Medicine, University Hospital Southampton NHS Trust, Southampton, UK; 4 National Institute for Health Research Biomedical Research Centre Southampton, University Hospital Southampton NHS Trust and University of Southampton, Southampton, UK; 5 Neonatology Hospital La Paz Institute for Health Research – IdiPAZ, (Universidad Autónoma de Madrid), Madrid, Spain; 6 The Department of Ophthalmology, Boston Children’s Hospital, Harvard Medical School, Boston, MA USA; 7 Department of Pediatrics, McMaster University, Hamilton, ON Canada; 8 Department of Pediatrics, Nuremberg General Hospital, Paracelsus Medical School, Nuremberg, Germany; 9 Réanimation Néonatale et Pédiatrique, CHU La Réunion, Saint-Pierre, France; 10 Department of Neonatal Medicine, University Hospital Southampton NHS Foundation Trust, Southampton, UK; 11 Neonatal Intensive Care Unit and EA7328-PACT, Necker-Enfants Malades University Hospital, Paris, France; 12 CNRC, Department of Pediatrics, Baylor College of Medicine, Houston, TX USA; 13 Division of Paediatric and Adolescent Medicine, Department of Neonatal Intensive Care, Ullevål, Oslo University Hospital, Oslo, Norway; 14 Neonatology, Instituto de Investigación Sanitaria del Hospital Universitario La Paz – IdiPAZ, (La Paz University Hospital – Universidad Autónoma de Madrid), Madrid, Spain; 15 Hans Christian Andersen Children’s Hospital, Department of Neonatology, Odense University Hospital, Odense, Denmark; 16 Department of Clinical Research, University of Southern Denmark, 5000 Odense, Denmark

## Abstract

**Abstract:**

Very preterm infants are at high risk of growth failure. Poor weight gain is a prominent risk factor for retinopathy of prematurity (ROP) and optimizing nutrition could potentially promote growth and reduce ROP. Most infants at risk of ROP need parenteral nutrition initially and studies of enhanced parenteral provision of lipids and amino acids have suggested a beneficial effect on ROP. Higher amino acid intake was associated with lower incidence of hyperglycemia, a risk factor for ROP. For very preterm infants, providing unpasteurized fortified raw maternal breast milk appears to have a dose-dependent preventive effect on ROP. These infants become deficient in arachidonic acid (ArA) and docosahexaenoic acid (DHA) after birth when the maternal supply is lost. Earlier studies have investigated the impact of omega-3 fatty acids on ROP with mixed results. In a recent study, early enteral supplementation of ArA 100 mg/kg/d and DHA 50 mg/kg/d until term equivalent age reduced the incidence of severe ROP by 50%.

**Impact:**

- Previous reviews of nutritional interventions to prevent morbidities in preterm infants have mainly addressed bronchopulmonary dysplasia, brain lesions and neurodevelopmental outcome. This review focusses on ROP.
- Neonatal enteral supplementation with arachidonic acid and docosahexaenoic acid, at levels similar to the fetal accretion rate, has been found to reduce severe ROP by 50% in randomized controlled trials.

## Introduction

Retinopathy of prematurity (ROP) is a neurovascular disease of preterm infants and a major cause of childhood blindness worldwide.^1^ Extremely preterm infant survival rates are increasing and, unfortunately, so have morbidities such as ROP. In Sweden, the proportion of screened infants treated for ROP increased from 2008 to 2017 and over time, more very immature infants and fewer with higher gestational age (GA) were treated.^2^ In the US, the incidence of ROP doubled from 2003 to 2019, and the increase was most pronounced in low-income areas.^3^

ROP has two phases. In the first phase, starting directly after birth, increased and fluctuating oxygenation, oxidative stress and inflammation, as well as insufficient nutrient supply contributes to post-natal weight loss and impaired retinal blood vessel growth.^4,5^ A sharp demarcation line between vascularized and avascular retina develops. Later, body weight gain velocity increases.^6^ At this stage, retinal vascularization either develops relatively normally or the second phase of ROP occurs, with extra-retinal neovascularisation and risk of retinal detachment. Low GA and poorly controlled oxygen supplementation are well-known risk factors for ROP. In addition, poor weight gain and low circulating concentrations of insulin-like growth factor-1 (IGF-1) are strong risk factors. Tools for the prediction of severe ROP based on these variables have been developed for very preterm infants and are used worldwide to optimize screening.^7–10^ Circulating IGF-1, mainly produced in the fetal/infant liver, is a signal of nutritional status and health. IGF-1 stimulates growth and maturation and acts as a permissive factor for VEGF-induced normal retinal vascularization. Serum IGF-1 levels are partly nutrition dependent but are also downregulated by infections, hypoxic episodes, corticosteroids and other factors. Measures to prevent ROP include optimizing nutrition and preventing inflammation to increase IGF-1 and improve growth.

The aim of this narrative review is to discuss nutritional interventions evaluated for their possible ability to reduce ROP.

## Methods

We searched PubMed for articles in English up to August 2023 addressing the association between nutrition and ROP.

### Nutritional interventions to prevent ROP

Parenteral nutrition (PN) nutrition is recommended for very preterm infants starting at birth. Minimal enteral feedings are provided in increasing amounts as tolerated. Fortified maternal breast milk is preferred, but donor human milk (DHM) or preterm formula (PF) is used when the mother’s own milk (MOM) is unavailable in sufficient amounts. This regimen follows the recommendations of the American Academy of Pediatrics and the European Society for Pediatric Gastroenterology Hepatology and Nutrition (ESPGHAN).^11,12^

Nutritional interventions to prevent ROP include early enhanced nutrition to increase IGF-1 levels and growth. We will address PN as well as alternative for enteral nutrition. We will also discuss trials evaluating parenteral and enteral supplementation with LCPUFAs and other supplements.

#### Enhanced nutrition

Several studies have shown a strong relationship between poor weight gain and ROP.^10,13,14^ Weight gain is multifactorial. Immaturity, infections, hypoxic episodes, and other factors affect nutrient assimilation and utilization and contribute to low circulating IGF-1 and poor growth. About half of neonatal growth retardation in preterm infants is estimated to be related to nutritional intake.^15^ It can be speculated that increased amounts of energy, fat, carbohydrates, and protein as well as improved compositon of provided nutrition might promote normal retinal growth and vascularization. This may lead to reduced avascular retinal area and in turn, the resulting hypoxia known to stimulate abnormal neovascularization.

PN and formulas for preterm infants lack the variety of bioactive factors normally transferred via the placenta and ingested in amniotic fluid and breast milk, which vary according to the developmental stage and needs of the fetus and newborn infant.

Nutritional regimes for very preterm infants over the past two decades have not eradicated postnatal growth failure. However, in more recent studies early postnatal weight loss has decreased and subsequent weight gain has increased.^6^ Studies on the association between higher nutrient intake and outcome have mainly addressed neurodevelopment. Two systematic reviews from 2022 reported that enhanced enteral but not parenteral nutrition had a positive impact on neurodevelopment.^16,17^ Unfortunately, in most studies the two routes of administration are not presented separately. A longer duration of PN has been associated with severe ROP.^18,19^ Inclusion of PN for more than 14 days as a variable in the algorithm of the DIGI-ROP clinical decision support tool improved ROP prediction.^20,21^ Interestingly, an inverse association between the amount of PN given in the second week of life and circulating IGF-1 has been reported in infants with GA between 24 and 32 weeks.^22^

In a meta-analysis of five studies, with different ROP definitions, initiation of parenteral lipids (≥ 1.5 g/kg/day within the first 24 h of birth) showed a favorable effect on ROP compared to lower initial doses and/or later initiation.^23^ Very low-quality evidence suggested, according to a Cochrane review, that higher amino acid intake in parenteral nutrition reduces ROP, but not severe ROP. It was pointed out that high amino acid intake may not be tolerated by all infants.^24^ In the Extremely Low Gestational Age Newborn (ELGAN) study of 1180 infants with GA < 28 weeks, total (parenteral and enteral) intake of calories, lipids, and carbohydrates in the lowest quartile but not protein on postnatal days 3, 7, 14, and 21 was associated with increased risk of ROP needing treatment to prevent blindness. A growth velocity in the lowest quartile was associated with an increased risk of any ROP.^25^ In that study the nutritional goals for protein and fats were reached, whilst those for carbohydrates and total energy were not.^25^ Higher energy intake days 7 to 27 was associated with less ROP but not severe ROP in the Extremely Preterm Infant in Sweden Study (EXPRESS).^26^ Whether infants with the lowest energy intake would tolerate increased intake and in doing so, reduce their ROP risk is unknown. Can et al. found that infants on aggressive PN had increased IGF-1 levels from the third postnatal week and less ROP compared to infants on conventional nutritional management. However, included infants were relatively mature with a mean GA of approximately 29 weeks and therefore at less risk of ROP than ELGANs.^27^

Oxidative stress is a major risk factor for ROP and shielding parenteral solutions from light reduces the amount of peroxides infused.^28^ No effect of this intervention on ROP was found in a small study of infants with GA < 37 weeks.^29^

##### Maternal and donor human milk and preterm formula

Breast milk is the preferred nutrition for all infants, especially those born very preterm.^12^ Unprocessed MOM contains nutrients and a variety of multifunctional bioactive factors involved in nutrient absorption, immune system maturation, antioxidant, and anti-inflammatory defense, gut microbiome establishment, food tolerability, and metabolism. However, energy and protein content is insufficient to sustain growth in very preterm infants, meaning that fortification is needed. Suboptimal early nutrition affects boys more than girls with respect to neurodevelopmental outcomes.^30^

Breast milk content is modified in relation to maternal nutrition and infant characteristics such as BW, GA, stage of lactation, and sex, among others.^31^ The mothers of boys produce milk richer in energy and lipid content than mothers of girls, indicating a natural personalized process.^32^

If sufficient MOM is not available, pasteurized DHM is recommended.^11^ DHM differs from MOM, as it usually comes from mothers of healthy, more mature infants at a different stage of lactation and can come from a single donor or be pooled from multiple donors. It contains less protein, energy, and fat than MOM.^33^ Exclusive DHM feeding compared to fresh preterm milk resulted in a lower intake of DHA (10.6 vs. 16.8 mg/d) and ArA (17.4 vs. 25.2 mg/d) and in lower total fat intake than recommended by the ESPGHAN (3.7 vs. 6.7 g/d).^34^ In addition, DHM had higher lipid peroxidation than both preterm and term breast milk.^34^ DHM is commonly exposed to multiple freeze–thaw cycles which affect content. For microbiological safety the milk is heated to 62.5 °C for 30 min (Holder pasteurization), which reduces or destroys several of the bioactive factors. Other sterilization methods affect bioactive proteins differently.^35^ Unprocessed MOM contains beneficial bacteria and maternal immune cells as well as a number of various hormones and factors with multiple physiologic functions, which are destroyed or reduced with pasteurization. One example is bile salt stimulated lipase, which is involved in the digestion of fat and inactivated by pasteurization. Fat absorption was reduced by approximately one-third in infants fed pasteurized compared to raw milk.^36^ Lactoferrin is a component of the immune system with anti-microbial as well as anti-inflammatory properties and acts against bacteria, viruses and fungi. It is found in various secretory fluids and is especially abundant in human colostrum and raw breast milk and reduced by approximately 80% with pasteurization.^37^ In a Cochrane review of enteral lactoferrin supplementation, some preventive effect on threshold ROP was found but with low certainty of evidence.^38^

In comparison with PF, MOM^39^ but not DHM^40^ protects against severe ROP. In a randomized trial in 243 preterm infants (< 30 weeks GA at birth) of DHM versus PF as substitutes for MOM when supply was insufficient, infants exclusively fed MOM (MOM group) were compared to infants who received MOM plus DHM from women who had preterm infants (DHM group) or MOM plus PF (PF group). All infants received a bovine milk-based fortifier. In the DHM group, 17% of infants switched to PF due to poor growth, compared to none of those receiving exclusively MOM. Proliferative ROP stage 3 was found in 5.6% of the MOM group compared to 19% in the DHM group and 14% in the PF group, suggesting a protective effect of MOM against severe ROP.^41^ In addition, the quantity of MOM but not DHM or PF correlated negatively with infection related events such as NEC and late-onset sepsis.^41^ Therefore, efforts to promote the provision of MOM to infants at risk for ROP might be the most important preventive intervention.

##### Long chain polyunsaturated fatty acids

LCPUFAs are structural and functional components of cell membranes. The brain is a lipid-rich organ. DHA and ArA are the principal, (highly conserved LCPUFA) components of brain lipids. ArA is especially enriched in endothelium and immune system membranes and is essential for vascularization, growth and other processes during development. ArA is abundant in the retina and in retinal and choroidal blood vessels^42^ and associated with growth and development in animals and humans, including preterm infants.^43^ DHA is abundant in neuronal tissue with the highest concentrations in retinal photoreceptor outer segments. Both fatty acids are the bases of a variety of bioactive metabolites involved in a variety of processes, such as metabolism, inflammation, and angiogenesis. An optimal balance between Omega-6 and omega-3 LCPUFA is important for proper function.

During gestation, the placenta selectively transfers certain fatty acids to the fetus. Among the LCPUFAs, the transfer of ArA and its omega-6 allies di-homo-gamma-linolenic acid (DHGLA), adrenic acid and docosapentaenoic acid is the most facilitated. Omega-3 DHA transfer is also favored but to a lesser extent. DHA precursors including eicosapentaenoic acid (EPA) as well as the ArA precursor linoleic acid (LA) and oleic acid are rejected by the placenta.^44^ While the proportion of ArA in umbilical cord blood fatty acids is constant and exceeds maternal values throughout gestation, the level of DHA increases above maternal levels from around 33 weeks, concomitant with increased lipid accumulation in the central nervous system and adipose tissue.^45^ Currently used parenteral soy- or olive- based lipid solutions have insufficient levels of DHA and ArA, but newer fish oil containing solutions contain more DHA and EPA although they were not developed specifically for preterm infants. In breast milk, DHGLA, ArA and adrenic acid have been found in proportions almost twice those of EPA and DHA.^44^ In addition, DHA and ArA levels in breast milk were linked and higher DHA levels were associated with higher ArA levels.^46^

Very preterm infants are born with low LCPUFA reserves and they accumulate a deficit in ArA and DHA in the neonatal period.^47^ At term equivalent age ArA and DHA content in adipose tissue, plasma and erythrocyte membranes is reduced and LA increased in these infants.^48^ While much research has focused on supplementation with omega-3 LCPUFAs to preterm infants, few studies have addressed the role of ArA in neonatal development. Scant evidence of this role has resulted in recommendations by the European Commission without a lower limit for ArA supplementation to infant formula, which has been questioned by experts in infant nutrition.^49^

There is evidence that girls and boys may have different LCPUFA requirements.^44^ LCPUFAs are prone to peroxidation and girls have been reported to have increased antioxidant activity and less oxidative stress than boys.^50^

##### LCPUFA and ROP

Clinical trials supplementing preterm infants with omega -3 fatty acids or pure DHA to prevent ROP have been somewhat inconsistent. In a mouse model of ROP, omega-3 LCPUFA supplementation suppressed TNFα and reduced pathologic retinal neovascularization.^51^

Parenteral administration of fish oil rich in omega-3 LCPUFAs has yielded inconsistent results. In a randomized study, infants receiving an intravenous fat emulsion containing fish oil had less ROP requiring laser treatment.^52^ In a cohort study and in an RCT, significantly fewer infants in the group that received fish oil developed any stage ROP.^53,54^ In extremely preterm infants with a median parenteral nutrition duration of 8 days no effect of fish oil on ROP incidence was observed.^55^ A systematic review and meta-analysis in 2017 concluded that the use of fish-oil lipid emulsions may reduce the incidence of severe ROP or the need for laser therapy in preterm infants.^56^ However, in 2019 a Cochrane review found no support for the use of fish oil containing lipid emulsions compared to non-fish oil emulsions to reduce ROP.^57^ Enteral DHA supplementation may reduce severe ROP.^58^

There is a delicate balance between different fatty acids in the body, and the provision of one LCPUFA might influence the concentrations of others, because the metabolic pathways of omega-3 and omega-6 LCPUFA are competitive. Administering omega-3 LCPUFAs to extremely preterm infants has resulted in increased levels of circulating DHA and EPA but also in reduced ArA levels.^55,59^ In a trial comparing PN with and without fish oil, lower serum fractions of ArA were associated with ROP.^60^ Interestingly, it has been demonstrated in one cohort, that higher mean daily serum levels of DHA during the first 28 postnatal days were associated with lower frequency of severe ROP even after adjustment for known risk factors, but only in those infants with sufficiently high ArA levels (mean daily minimum level of 7.8 to 8.3 molar percent).^61^

**Fig. 1:**
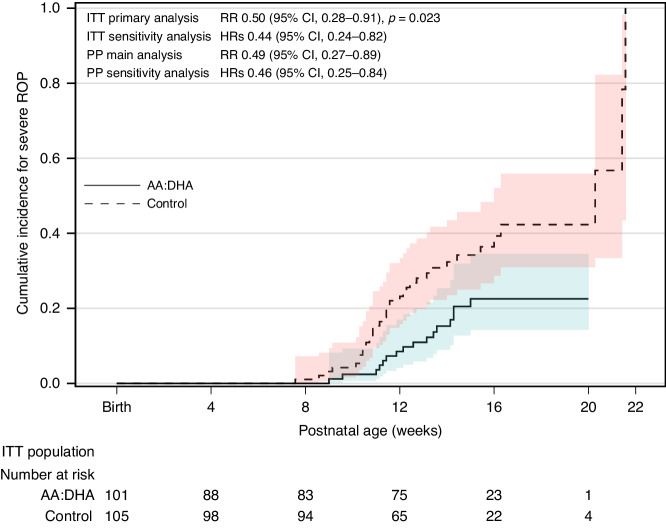
Cumulative incidence functions for severe retinopathy of prematurity (intention to treat population) (Hellström et al. JAMA Pediatr 175, 359–367 (2021)).

In the Mega Donna Mega trial, infants with GA < 28 weeks were randomized to receive enteral ArA (100 mg/kg/d) and DHA (50 mg/kg/d) or no supplementation from within 3 days after birth until 40 weeks’ PMA. The supplement increased circulating fractions of both ArA and DHA and reduced severe ROP by 50% (Fig. 1).^62^ In a later Norwegian study of infants with GA < 29 weeks, a similar intervention demonstrated reduced incidence of severe ROP from 12.7% in the control group to 5.4% in the supplemented group. This difference was not statistically significant, possibly due to the low rate of severe ROP in the study population. In the Norwegian study, supplemented infants had less oxygen demand and fewer days with respiratory support than controls.^63^

Therefore, enteral supplementation with both ArA and DHA appears to be a simple intervention that can prevent ROP. The optimal dosage has not been determined, though it has been proposed that the ratio of ArA/DHA should be higher than 2/1, thus reflecting fetal levels, especially in infants with GA < 29 weeks since fetal ArA levels are constant throughout gestation while DHA levels increase from around 33 weeks^45,64^. In cord plasma, the ratio of ArA/DHA has been reported to decrease from 4.9 at 24–27 weeks to 2.5 at term.^45^ ESPGHAN recommends the provision of 30–65 mg/kg/d of DHA if ArA intake is sufficient and 30–100 mg/kg/d of ArA to preterm infants with BW <1800 g.^12^ Overall, whether LC-PUFAs affect the incidence or severity of ROP requires further investigation.

##### Micronutrients

Vitamin E is a strong antioxidant that has been studied decades ago for its proposed preventive effect on ROP. However, trials of vitamin E supplementation in the 1990s were ended due to reports of adverse events such as necrotizing enterocolitis, sepsis and intraventricular hemorrhage as reviewed by Ogihara.^65^ Few recent studies are available, but in 2021 a Mexican RCT reported lower levels of oxidative damage markers, increased antioxidant capacity, and a reduced ROP rate without adverse effects in infants who were orally supplemented with 25 IU vitamin E compared to placebo.^66^

Vitamin A downregulated VEGF and reduced neovascularization in a rat model of ROP.^67^ Of two recent meta-analyses of studies on vitamin A supplementation to preterm infants with BW < 1500 g, one found a preventive effect while the other found no effect on ROP.^68,69^

Zinc is a trace element involved in growth, immune defense, and other functions. Very preterm infants start out with a smaller zinc pool than term babies and zinc content in breast milk may not be sufficient to match fetal accretion rates. Enteral zinc supplementation resulted in a non-significant reduction in ROP in the one study analysed in a Cochrane review.^70^

The carotenoids lutein and zeaxanthin are antioxidants transferred to the fetus during the third trimester. Supplementation of these carotenoids to the mother reduced retinal avascular area and neovascularization in a mouse model of ROP.^71^ In humans, postnatal administration did not reduce threshold ROP but the progression rate from early stages to threshold ROP was reduced by 50%.^72^

##### Blood glucose management

Transient hyperglycemia, related to insulin resistance and excessive glucose infusion rates in parenteral nutrition, is a common complication of prematurity. It is associated with increased morbidity and mortality in very preterm infants, in particular severe ROP, after adjustments for its main risk factors.^73,74^ Hyperglycemia also impairs retinal neurovascular development in neonatal animal models.^75,76^ The impact of strategies to avoid or reduce hyperglycemia on preterm birth-associated morbidity, including ROP, has not been evaluated as a primary outcome in clinical trials. However, published data support limiting the frequency and intensity of hyperglycaemic episodes in very preterm infants, as the combined severity and duration of hyperglycemia in the first three weeks of life is more important than the average or maximum value of glycemia in determining the risk of severe ROP.^74^ In line with these observations, the ESPGHAN recommends that hyperglycemia > 8 mmol/L (145 mg/dL) should be avoided and that repeated blood glucose levels >10 mmol/L (180 mg/dL) should be treated with insulin therapy if a reasonable adjustment of the glucose infusion rate has been insufficient.^77^

A recent review and meta-analysis concluded that insulin therapy may not improve outcomes of very preterm infants with hyperglycemia^78^ The gluconeogenesis initiated within the first days of life in preterm infants on PN is not regulated by either glucose or insulin.^79^ Early enteral feeding and early provision of amino acids have been shown to reduce hyperglycemia in very preterm infants and may promote growth and possibly reduce ROP.^24,80,81^

## Discussion

While several risk factors for ROP are linked to nutritional practices, such as impaired postnatal growth and inadequate nutrient intake or low plasma IGF levels, few nutritional interventions have suggested a preventive effect on ROP. When a nutritional intervention is implemented its effects may differ depending on whether the subjects treated are deficient, at risk of deficiency, or not deficient in the targeted nutrient. The definition of deficiency and standard requirements for many nutrients are poorly known in very preterm infants and may vary according to factors such as gestational age, sex, intrauterine growth restriction, or metabolic stress; this is a barrier to demonstrating the effectiveness of nutrition interventions. In addition, as ROP, like most complications of prematurity, is a multifactorial disease, the effectiveness of nutritional interventions in preventing ROP will depend on the balance of exposure to its different risk factors within an individual NICU, and, therefore on management practices, including saturation targets policy, parenteral and enteral nutrition protocols, availability of lipid emulsions, glycaemic management or transfusion policy.

The reason for parenteral nutrition as a risk factor for ROP is currently unknown but may be linked to inadequate composition of nutrients, lack of proper enteral stimulation and of initial hepatic passage.

The most important nutritional intervention to reduce ROP is to encourage and support the provision of MOM. The establishment of optimal milk production after extremely preterm birth is challenging and milk production is often delayed. Prenatal information about MOM as “a medicine” for preterm infants and its dose-dependent positive effects on the infant may help to motivate mothers to initiate lactation.^82^ In the NICU, proper lactation support with early initiation of breast milk pumping, frequent pumping and kangaroo care may help to establish lactation.

Since very preterm infants miss the third trimester transfer of LCPUFAs, micronutrients, and other factors from the mother, supplementation to correct or prevent deficiencies may reduce ROP and other morbidities. For the newborn, ArA and other omega-6 fatty acids are essential drivers of organogenesis, growth, and development, and the importance of a proper ArA/DHA balance has become increasingly recognized.^44^ The ArA needs of preterm infants are not well known and at present doses of 30–100 mg/kg/d per day of ArA and 30–65 mg/kg/d of DHA are recommended.^12^ The commonly used parenteral lipid emulsion Clinoleic® contains insufficient amounts of ArA and DHA.^83^ In the Mega Donna Mega trial, enteral administration of an additional 100 mg/kg/d of ArA and 50 mg/kg/d DHA reduced severe ROP by 50%, which makes it a promising, easily administered nutritional intervention to prevent ROP.^52^ In future studies, different doses may be tested. The ArA/DHA ratio in umbilical cord blood decreases from 24–27 weeks to term since the transfer of DHA via the placenta increases in the third trimester.^45^ Boys and girls appear to have different needs and react differently to nutrition in general and also with regard to LCPUFA supplementation.^84–86^ The potential impact of adjustments of doses and exposures in relation to PMA and sex needs further study.

## Conclusions

For the newborn preterm infant, the most important nutritional intervention is early enteral feeding of MOM, which appears to have a dose-dependent protective effect of ROP and other morbidities. Significant support is needed to help mothers of extremely preterm infants to initiate and sustain lactation. Optimizing lipids and amino acids in parenteral nutrition may have a beneficial effect on ROP. Supplementation with enteral ArA and DHA from birth to 40 weeks has the capacity to significantly reduce severe ROP.

## Data Availability

Data sharing not applicable to this article as no datasets were generated or analysed during the current study.
